# A brief report: absence of elevated circulating NETosis markers in long-term long COVID

**DOI:** 10.3389/fcimb.2026.1920533

**Published:** 2026-09-08

**Authors:** Bart Humer, Hande Eyisoylu, Julia C. Berentschot, L. Martine Bek, Chantal A. Boly, Merel E. Hellemons, Moniek P.M. de Maat

**Affiliations:** 1 Department of Immunology, Erasmus Medical Center, Rotterdam, Netherlands; 2 Department of Hematology, Erasmus Medical Center, Rotterdam, Netherlands; 3 Department of Respiratory Medicine, Erasmus Medical Center, Rotterdam, Netherlands; 4 Department of Intensive Care, Erasmus Medical Center, Rotterdam, Netherlands; 5 Department of Intensive Care, Elisabeth-TweeSteden Hospital, Tilburg, Netherlands

**Keywords:** biomarkers, inflammation, Long COVID, NETosis, neutrophils, post-COVID, post-acute sequalae of SARS-CoV-2 infection

## Abstract

**Background:**

Neutrophil extracellular trap (NET) formation (NETosis) has been proposed as a contributor to the pathophysiology of Long COVID (LC). However, it remains unclear whether markers of systemic NETosis remain elevated in individuals with persistent symptoms years after the initial infection.

**Methods:**

To assess NETosis, we quantified MPO-DNA, Histone DNA, and Citrullinated H3 levels in the plasma of 51 patients with prolonged LC (mean disease duration of three years), and compared them with 52 age- and sex-matched healthy controls.

**Results:**

No significant differences were observed between participants with LC and healthy controls for any of the three NETosis markers. Hierarchical clustering reveals no specific subgroups in the patient group. Furthermore, NETosis marker levels were not associated with overall symptom burden or individual symptom domains.

**Conclusions:**

In this cohort of individuals with long-term LC, we found no evidence of persistent systemic NETosis. These findings suggest that elevated circulating NETosis markers are not a universal feature of long-term LC and may indicate that neutrophil activation observed during acute or early post-acute disease does not persist in long-term disease.

## Introduction

Long COVID (LC), also referred to as post-COVID-19 or post-acute sequelae of SARS-CoV-2 infection, is characterized by persistent symptoms more than 3 months after the acute SARS-COV-2 infection, which may last for years (Greenhalgh et al., 2024). While the exact pathophysiological mechanisms are unknown, consensus is reached on main hypotheses involving immune dysregulation, chronic inflammation, endothelial dysfunction, viral persistence, and autoimmunity (Davis et al., 2023).

Within this framework of persistent immune dysregulation, neutrophil extracellular traps (NETs) have been proposed as a contributor to LC pathogenesis (Shafqat et al., 2023; Monsalve et al., 2025; Serrano-Gonzalo et al., 2025). NETs are structures composed of intracellular granules, DNA, and histones released by activated neutrophils (Mutua and Gershwin, 2021). These structures are vital during an innate immune response as they capture viral, fungal, bacterial, and protozoal pathogens and modulate immune cell function (Wang et al., 2024).

However, NETs are non-specific, enabling collateral damage by acting as damage associated patterns (DAMPs), which can either exacerbate or initiate inflammatory responses (Mutua and Gershwin, 2021). During acute COVID-19, excessive NET formation has been implicated in thromboinflammation and tissue injury (Gillot et al., 2021). Several studies have reported elevated markers of NETosis during acute infection and, in some cohorts, during the early post-acute phase (Ackermann et al., 2021; Janiuk et al., 2021; Vitkov et al., 2022).

Yet, data on NETosis in individuals with long-standing LC and its contribution to LC pathophysiology is lacking. We hypothesize that long-term LC is partly characterized by persistent NETosis, reflecting sustained immune activation. Accordingly, we expect to observe increased levels of NETosis markers in individuals with long-term LC.

The aim of the present study was therefore to assess circulating NETosis markers in the plasma of patients with long-term LC compared to healthy controls.

## Methods

### Study population

#### Study design and participants

In this study, we recruited participants with severe LC who were age- and sex-matched with convalescent healthy controls (HC). Participants with LC were 18–65 years of age, experienced COVID-19, had an LC diagnosis for ≥ 6 months, reported post-exertional malaise (PEM), and reported an overall functioning of ≤70% compared to their pre-COVID-19 health status. HC experienced COVID-19 without developing LC symptoms. Participants were excluded if they had a recent (<3 months) COVID-19 reinfection or vaccination, used anti-inflammatory medication, or experienced any other conditions that could elucidate symptoms. Both HC and LC participants had experienced mild acute COVID-19 (self-reported), with no participants requiring ICU-care during the acute phase. The Medical Ethics Committee of Erasmus MC approved the study (NL-005067).

Participants were included between December 2023 and January 2024. Blood samples were obtained from all participants and they completed online questionnaires comprising patient-reported outcome measures (PROMs) to assess their health status. Demographic and clinical data were collected for age, sex, body mass index, smoking status, employment status, leisure-time physical activity, pre-existing comorbidities, and COVID-19-related characteristics. The iMTA productivity cost questionnaire and the Saltin-Grimby Physical Activity Level Scale questionnaire were used to obtain information regarding employment status and work capacity, and leisure-time physical activity, respectively, both pre- and post-development of LC (Bouwmans et al., 2015; Grimby et al., 2015). Multiple PROMs were administrated; the frequency and severity of PEM was assessed by DePaul symptom questionnaire short form (Cotler et al., 2018; Kielland et al., 2023), symptoms of postural tachycardia syndrome (POTS) with the Malmo POTS score questionnaire (Spahic et al., 2023), fatigue with fatigue assessment scale (de Kleijn et al., 2011), dyspnea with the medical research dyspnea scale (Williams, 2017), cognitive failures with the cognitive failure questionnaire (Broadbent et al., 1982), and health-related quality of life with the 5-level EuroQoL-5D (EQ-5D-5L) questionnaire (EuroQol, 1990). In addition, a symptom questionnaire assessed 32 symptoms that newly occurred or worsened after acute COVID-19. Overall functioning compared with pre-COVID-19 health was assessed using a 0-100% scale. Data were stored in Castor Electronic Data Capture system (Castor EDC, Amsterdam, the Netherlands). The timing of the SARS-CoV-2 infection causing LC (LC group) or first infection (Cc group) was categorized into Wuhan-Hu-1 (February 2020─mid-February 2021), Alpha (mid-February 2021─June 2021), Delta (July 2021─end-December 2021), and Omicron (end-December 2021─present) variants, aligning with the timeframes in which these variants were dominant in the Netherlands.

### Measurement of NETosis markers

Blood was drawn into sodium citrate tubes (final concentration 0.105M) using a Vacutainer System (Beckton, Dickinson and Company, Plymouth, UK). The blood was centrifuged at 2500g for 10 min at room temperature and plasma was stored in aliquots at − 80 °C until analysis.

We quantitatively measured histone-DNA complexes levels in plasma by using a commercial human cell death ELISA kit (Cell Death Detection ELISAPLUS, Cat. No. 11 920 685 001; Roche Diagnostics GmbH, Mannheim, Germany), and CitH3 levels by the ELISA kit Citrullinated Histone H3 (Clone 11D3) (Cayman CHMICAL, Cat. No. 501620, Ann Arbor, MI, USA). For the MPO-DNA complexes ELISA, we adapted the commercial human cell death ELISA kit (Cell Death Detection ELISAPLUS, Cat. No. 11 920 685 001; Roche Diagnostics GmbH, Mannheim, Germany). We employed an anti-MPO monoclonal antibody (clone 4A4, Isotype IgG2b, Sanbio B.V., #0400–002) as the capturing antibody. Plasma samples from participants were added in combination with peroxidase-labeled anti-DNA monoclonal antibody (Component No. 2 of the Cell Death Detection ELISA kit; Roche, #11–774-425–002) in the solution. Absorbance was measured at 450 nm using a Biotek Synergy HT plate reader, with a reference filter set to 490 nm. As a control, we induced NETosis with PMA in neutrophils isolated from healthy volunteers using Lymphoprep (Prod. No. 1114544, Serumwerk Bernburg AG, Oslo, Norway). Cells were stored as aliquots in fetal calf serum until MPO-DNA complex analysis. Results were expressed in arbitrary units.

### Statistical analysis

Differences between groups were evaluated using Mann–Whitney U tests in PRISM 8.0. Correlations between marker levels and PROMs were assessed using Spearman correlation coefficients. Unsupervised hierarchical cluster analysis, correlation plots and forest plot, were performed using the pheatmap and ggplot2 package in R studio. Descriptive statistics were obtained using the stats package in R. A two-sided p-value < 0.05 was considered statistically significant.

## Results

### Participant characteristics

Data from 51 LC patients and 52 HC were analyzed (**Table 1**). The median age of the LC population was 40 (IQR 34-49) years and 27 (53%) were female. Median LC duration was 3.0 (1.9-3.7) years. No significant demographic differences were observed between LC and HC groups. A higher incidence of neurodevelopmental or mental health conditions were reported in LC group compared with HC groups (21 [42%] vs 7 [14%], p =0.004, respectively). Unemployment, medication usage, and lower physical activity levels were more common in the LC group as opposed to HC group.

**Table 1:** Characteristics of patients with long COVID and healthy controls.

	N	LC group (n = 51)	HC group (n = 52)	P value
LC duration^a^ (years)	51	3.0 (1.9-3.7)	NA	
Age (years)	─	40 (34–49)	41 (32–49)	0.80
Sex, female	─	27 (53%)	27 (52%)	1.00
BMI (kg/m^2^)	100	24 (22–26)	24 (22-27)	0.69
Physical activity level^b^	100			
Pre-COVID-19				0.57
Inactive		1 (2%)	2 (4%)	
Light		8 (16%)	12 (24.5%)	
Moderate		24 (48%)	23 (47%)	
Vigorous		17 (34%)	12 (24.5%)	
Post-COVID-19				**<0.001**
Inactive		31 (62%)	1 (2%)	
Light		19 (38%)	15 (31%)	
Moderate		0 (0%)	21 (43%)	
Vigorous		0 (0%)	12 (24%)	
Smoking	100			0.69
Never		35 (70%)	31 (62%)	
Former		13 (26%)	16 (32%)	
Current		2 (4%)	3 (6%)	
Employment	100			
Employed pre-COVID-19		47 (94%)	47 (94%)	1.00
Post-COVID-19				**<0.001**
Employed		21 (41%)	48 (98%)	
Unchanged hours		0 (0%)	48 (98%)	
Reduced hours due to LC		20 (40%)	NA	
Unemployed due to LC		26 (52%)	NA	
Homemaker		2 (4%)		
Student^c^		2 (4%)	1 (2%)	
Comorbidities	100			
Obesity (BMI ≥ 30 kg/m^2^)		7 (14%)	3 (6%)	0.32
Pulmonary disease		13 (26%)	6 (12%)	0.12
Cardiovascular disease		1 (2%)	0 (0%)	1.00
Gastrointestinal or liver disease		1 (2%)	0 (0%)	1.00
Mental health condition		11 (22%)	2 (4%)	**0.01**
Autoimmune disease		2 (4%)	0 (0%)	0.49
**COVID-19 characteristics**				
SARS-CoV-2 infection^d^	─			**0.04**
Wuhan-Hu-1		29 (57%)	18 (35%)	
Alpha		0 (0%)	2 (4%)	
Delta		7 (14%)	5 (10%)	
Omicron		15 (29%)	27 (52%)	
Hospitalization	100	0 (0%)	1 (2%)	1.00
Number of known SARS-CoV-2 infections	100			0.08
1		23(46%)	34 (68%)	
2		21 (42%)	12 (24%)	
≥ 3		6 (12%)	4 (8%)	
Time since latest infection (years)		1.9 (1.2-2.0)	1.6 (1.0-2.2)	0.68
Number of vaccinations	100			
0		1 (2%)	3 (6%)	0.76
1		2 (4%)	2 (4%)	
2		13 (26%)	14 (28%)	
≥ 3		34 (68%)	31 (62%)	
Time since latest vaccination (years)		1.9 (1.2-2.0)	1.9 (1.2-2.0)	0.68
**Medication use** ^e^	100			
PEM-directed therapies		6 (12%)	0 (0%)	**0.03**
POTS-directed medication		8 (16%)	0 (0%)	**0.006**
Antidepressants or neuropathic pain medication		14 (28%)	1 (2%)	**<0.001**
Benzodiazepines		7 (14%)	1 (2%)	0.06
Anti-allergy medication		8 (16%)	4 (8%)	0.36
Respiratory medication		7 (14%)	1 (2%)	0.06
Supplements		32 (64%)	0 (0%)	**<0.001**

Data are presented as median (IQR) or n (%). Adjusted N is presented for variables with missing data. P-values are obtained using the Mann-Whitney U test for continuous variables and the Chi-squared test or Fisher Exact test for categorical variables, as appropriate. A statistically significant p-value (p<0.05) is indicated in bold. LC, long COVID; HC, healthy controls; PEM, post-exertional malaise; POTS, postural orthostatic tachycardia syndrome; PTSD, post-traumatic stress disorder.

^a^ Disease duration was calculated as the time between the SARS-CoV-2 infection causing LC and the study visit.

^b^ Pre-COVID-19 leisure time physical activity level was measured with the Saltin–Grimby Physical Activity Level Scale questionnaire.

^c^ Includes students with or without a paid job.

^d^ Based on the timing of infection during periods in which these SARS-CoV-2 variants were dominant in the Netherlands, corresponding to the infection preceding LC in the LC group and to the first infection in the Cc group.

^e^ Categories include PEM-directed therapies (e.g., naltrexone, aripiprazole), POTS-directed medication (e.g., propranolol, metoprolol), antidepressants or neuropathic pain medication (e.g., citalopram, fluoxetine, fluvoxamine, amitriptyline, mirtazapine), benzodiazepines (e.g., temazepam, diazepam), anti-allergy medication (e.g., loratadine, cetirizine), respiratory medication (e.g., bronchodilators), and the use of any supplement.

**Supplementary Table 1** and **S2** present data from all administered PROMs. Overall, patients reported severe illness; all experienced ≥5 newly developed symptoms (median 15 [12-17] symptoms) and they reported 20% (15–29) functioning of pre-COVID-19 health status.

### NETosis markers are not elevated in Long COVID

**Figure 1:**
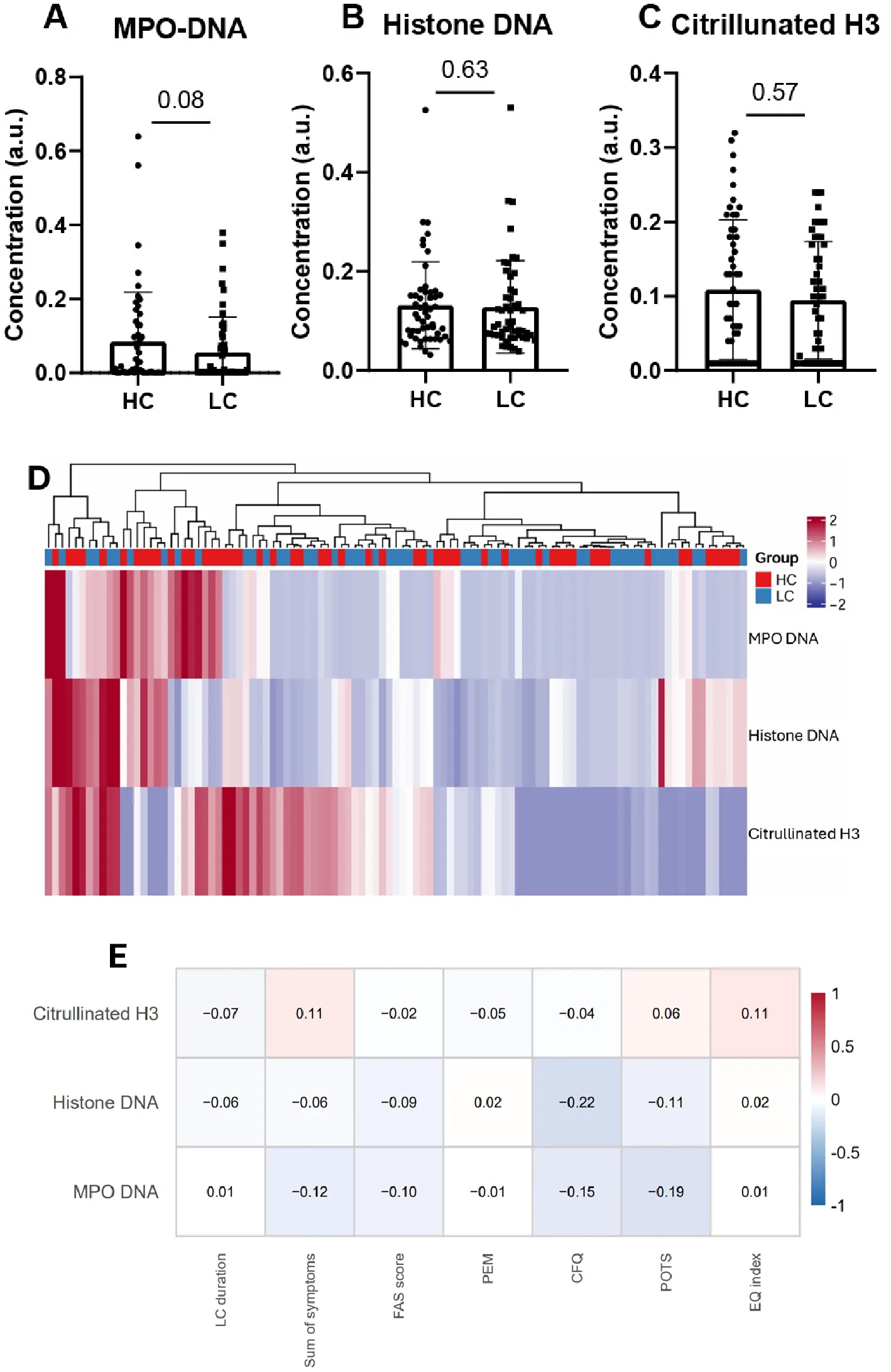
Plasma levels of NETosis markers MPO-DNA **(A)**, Histone-DNA **(B)**, and Citrullinated H3 **(C)** are presented as bar graphs. **(D)** shows a log-scaled hierarchical clustered heatmap to identify potential subgroups. **(E)** is a correlation plot of NETs plasma values with duration of long COVID, number of new symptoms, fatigue (FAS score), PEM, cognitive failures (CFQ), POTS, and quality of life (EQ index). Spearman’s rank correlation coefficient is shown. A Mann Whitney U test was used to compare groups, HC, healthy controls; LC, long COVID.

Plasma levels of MPO-DNA (**Figure 1A**, p=0.08),histone-DNA (**Figure 1B**, p=0.63) and citrullinatedhistone H3 (H3) (**Figure 1C**, p=0.57) did not differsignificantly between LC and HC (**Supplementary Table 3**). Similarly, hierarchical clustering analysis did not identify distinct subgroups withineither the LC or HC cohorts that would allow for further patient stratification (**Figure 1D**). No significant associations were observed between NETosis marker concentrations and symptom severity measures and core LC symptoms (**Figure 1E**). Multivariable linear regression models assessing the association of LC status with plasma MPO-DNA, histone-DNA, and citrullinated H3 levels did not reveal any significant association between LC status and measured markers (**Supplementary Figure 1**, **Supplementary Table 4**).

## Discussion

To our knowledge, we are the first to present NETosis markers in a well characterized LC cohort with a median disease duration of three years. We found no evidence of elevated systemic NETosis, based on three circulating NETosis markers in LC patients compared with age- and sex-matched healthy controls. Additionally, hierarchical clustering analysis did not identify any patient subgroups with potential stratification value. These findings differ from those reported during acute COVID-19 and contrast with studies proposing NETosis as a key mechanism underlying the development and persistence of LC (Shafqat et al., 2023; Monsalve et al., 2025; Serrano-Gonzalo et al., 2025).

While NETs are often discussed as a potential contributor to LC in literature, a consistent body of high-quality evidence demonstrating their involvement in prolonged LC is still lacking. Current literature reports increased neutrophil activation in children and young adults; however, disease duration was not reported (Steifman et al., 2026). Another study also found elevated serum levels of MPO-DNA complexes in LC, although the median time between infection and measurement was 217 days compared to non-convalescent controls (Monsalve et al., 2026). Additionally, elevated MPO–DNA complexes have been reported in individuals with a persistent symptom one month after acute COVID-19 infection, suggesting a possible association with very early LC onset (Krinsky et al., 2023). However, this may also reflect residual post-acute inflammatory activity from the acute infection rather than an LC–specific process. To date, no studies have examined correlations between NET markers and clinical symptoms in prolonged LC. These differences may be explained by our later sampling timepoint and the use of infection-recovered controls rather than pre-pandemic healthy controls.

Several limitations should be acknowledged. The cross-sectional nature of our study limits our ability to determine whether NET formation may occur at different stages of LC. Longitudinal sampling and functional NETosis assays are required to further elucidate the potential contribution of NETosis to LC pathophysiology. Additionally, indirect markers associated with NET-related processes, including endothelial injury markers, were not evaluated in the current study.

## Conclusion

Three circulating NETosis markers were similar in 51 individuals with LC compared with 52 healthy controls. This study found no evidence of NETosis abnormalities in patients with severe LC at a median follow-up of 3 years. These results argue against persistent systemic NETosis as a general characteristic of long-duration LC and suggest that systemic neutrophil activation observed during acute disease may not persist into the chronic phase. However, future longitudinal studies incorporating a broader panel of NETosis markers are warranted to enable more definitive conclusions.

## Data Availability

The datasets presented in this article are not readily available because the data that support the findings of this study are available from the corresponding author upon reasonable request. Requests to access the datasets should be directed to b.humer@erasmusmc.nl.
