# Supplementary material for: A brief report: absence of elevated circulating NETosis markers in long-term long COVID
Source: Front Cell Infect Microbiol. 2026 Sep 8;16:1920533. doi: 10.3389/fcimb.2026.1920533 (PMC13593446; doi:10.3389/fcimb.2026.1920533)
Supplement: Supplementary file 1 [file DataSheet1.docx]

**Supplementary Figure 1.** Forest plots showing adjusted regression coefficients (β) with 95% confidence intervals (CI) from multivariable linear regression models assessing the association of clinical and treatment-related factors with plasma MPO-DNA, histone-DNA, and citrullinated H3 levels. Models were adjusted for LC status, BMI, mental health, number of known SARS-CoV-2 infections, % functioning relative to pre-COVID-19, and medication usage. Dots represent the estimated regression coefficient, and horizontal lines indicate the 95% CI. The dashed vertical line represents no association (β = 0). None of the assessed covariates showed a significant association with MPO-DNA, histone DNA, or citrullinated H3.

**Supplemental Table 1**. Overview of health outcomes.

|  | **LC group** | **HC group** | **P value** |
| --- | --- | --- | --- |
| PEM |  |  |  |
| PEM, self-reported | 51 (100%) | 0 (0%) | **<0.001** |
| PEM, DSQ-PEM-sf screening | 49 (98%) | 2 (4%) | **<0.001** |
| Functioning compared to pre-COVID-19 health (%) | 20 (15-29) | 100 (100-100) | **<0.001** |
| Symptoms^a^ |  |  |  |
| Number of new symptoms | 15 (12-17) | 0 (0-1) | **<0.001** |
| Fatigue | 49 (98%) | 5 (10%) | **<0.001** |
| Impaired fitness | 49 (98%) | 2 (4%) | **<0.001** |
| Concentration problems | 48 (96%) | 2 (4%) | **<0.001** |
| Sensory overload | 47 (94%) | 6 (12%) | **<0.001** |
| Word-finding difficulties | 42 (84%) | 3 (6%) | **<0.001** |
| Memory problems | 41 (82%) | 4 (8%) | **<0.001** |
| Sleep disturbances | 39 (78%) | 3 (6%) | **<0.001** |
| Muscle pain | 38 (76%) | 1 (2%) | **<0.001** |
| Headache | 36 (72%) | 1 (2%) | **<0.001** |
| Palpitations | 36 (72%) | 1 (2%) | **<0.001** |
| POTS^b^ | 23 (45%) | 3 (6%) | **<0.001** |
| Positive NASA lean test | 14 (28%) | 3 (6%) |  |
| Positive tilt-table test | 12 (24%) | 0 (0%) |  |
| MAPS score >43 | 33 (66%) | 0 (0%) | **<0.001** |
| MAPS score total score | 51 (38-70) | 3.0 (0.0-8.0) | **<0.001** |
| Fatigue |  |  |  |
| Fatigue (FAS >22) | 48 (96%) | 9 (18%) | **<0.001** |
| FAS total score | 40 (35-43) | 18 (14-20) | **<0.001** |
| Cognitive failures |  |  |  |
| Cognitive failures (CFQ >43) | 30 (60%) | 3 (6%) | **<0.001** |
| CFQ total score | 49 (37-59) | 21 (13-29) | **<0.001** |
| Dyspnea |  |  |  |
| mMRC grade ≥1 | 31 (62%) | 0 (0%) | **<0.001** |
| HRQoL |  |  |  |
| EQ-5D index | 0.41 (0.24-0.57) | 1.0 (0.89-1.0) | **<0.001** |
| EQ-VAS | 29 (20-40) | 85 (80-90) | **<0.001** |

*Data are presented as median (IQR) or n (%) for 50 patients with long COVID and 49 healthy controls, except for self-reported PEM and the NASA lean test, which were obtained from all participants (51 patients with LC and 52 healthy controls). P-values are obtained using the Mann-Whitney U test for continuous variables and the Fisher Exact test for categorical variables. LC, long COVID; HC, healthy controls; PEM, post-exertional malaise; DSQ-PEM-sf,* *DePaul symptom questionnaire short form; POTS, postural orthostatic tachycardia syndrome; MAPS score, Malmo POTS symptom score; FAS, fatigue assessment scale; mMRC, modified medical research council dyspnea scale; CFQ, cognitive failures questionnaire; HRQoL health-related quality of life; EQ-5D, 5-level EuroQoL-5D questionnaire; EQ-VAS, EQ-visual analogue scale.*

*^a^ Top ten most frequently reported symptoms. Frequencies of all 32 assessed symptoms are presented in Supplemental Table S2.*

*^b^ POTS was indicated either by a heart rate increase of* ≥*30 bpm or exceeding 120 bpm during the NASA lean test, a self-reported previous positive tilt-table test, or use of POTS-directed medication.*

**Supplementary Table S2.** Prevalence of 32 symptoms in patients with long COVID and convalescent controls.

|  | **LC group** | **HC group** |
| --- | --- | --- |
| Fatigue | 49 (98%) | 5 (10%) |
| Impaired fitness | 49 (98%) | 2 (4%) |
| Concentration problems | 48 (96%) | 2 (4%) |
| Sensory overload | 47 (94%) | 6 (12%) |
| Word-finding difficulties | 42 (84%) | 3 (6%) |
| Memory problems | 41 (82%) | 4 (8%) |
| Sleep disturbances | 39 (78%) | 3 (6%) |
| Muscle pain | 38 (76%) | 1 (2%) |
| Headache | 36 (72%) | 1 (2%) |
| Palpitations | 36 (72%) | 1 (2%) |
| Dyspnea | 35 (70%) | 0 (0%) |
| Balance problems | 34 (68%) | 1 (2%) |
| Muscle weakness | 33 (66%) | 1 (2%) |
| Tingling/pain in extremities | 30 (60%) | 2 (4%) |
| Stool problems | 30 (60%) | 0 (0%) |
| Chest pain | 29 (58%) | 1 (2%) |
| Vision problems | 27 (54%) | 2 (4%) |
| Tinnitus | 27 (54%) | 5 (10%) |
| Somberness | 24 (48%) | 3 (6%) |
| Joint pain | 20 (40%) | 3 (6%) |
| Food hypersensitivity | 19 (38%) | 0 (0%) |
| Nausea | 18 (36%) | 0 (0%) |
| Hoarseness | 15 (30%) | 0 (0%) |
| Skin rash | 16 (32%) | 2 (4%) |
| Cough | 15 (30%) | 1 (2%) |
| Anxiety | 14 (28%) | 0 (0%) |
| Anosmia | 12 (24%) | 4 (8%) |
| Ageusia | 10 (20%) | 2 (4%) |
| Decreased appetite | 7 (14%) | 0 (0%) |
| Phlegm | 7 (14%) | 0 (0%) |
| Hearing problems | 5 (10%) | 2 (4%) |
| Miction problems | 4 (8%) | 0 (0%) |

Data are presented as number (%) for 50 patients with LC and 49 healthy controls. LC, long COVID; HC, healthy controls.

**Supplementary Table S3.** Descriptive statistics of individual plasma markers.

| **Marker** | **HC Median [95% CI]** | **LC Median [95% CI]** | **Rank Biserial Correlation [95% CI]** | **P Value** |
| --- | --- | --- | --- | --- |
| **MPO-DNA** | 0.02 [0–0.08] | 0 [0–0.02] | 0.196 [-0.024–0.408] | 0.08 |
| **Histone DNA** | 0.11 [0.09–0.14] | 0.09 [0.08–0.12] | 0.056 [-0.162–0.278] | 0.63 |
| **Citrullinated H3** | 0.09 [0.05–0.14] | 0.09 [0.03–0.12] | 0.07 [-0.154–0.279] | 0.57 |

Data are presented for 51 patients with LC and 52 healthy controls. LC, long COVID; HC, healthy controls.

**Supplementary Table S4.** Descriptive statistics of adjusted regression coefficients (β).

| **Marker** | **Predictor** | **β [95% CI]** | **p value** |
| --- | --- | --- | --- |
| **Citrullinated H3** | HCvsLC | -0.042 (-0.114, 0.030) | 0.254 |
|  | BMI | -0.003 (-0.010, 0.003) | 0.295 |
|  | COVID-19 infections (n) | -0.003 (-0.036, 0.031) | 0.878 |
|  | Medication usage | 0.003 (-0.011, 0.016) | 0.713 |
|  | Function | -0.000 (-0.000, 0.000) | 0.543 |
|  | Mental health | -0.010 (-0.086, 0.065) | 0.786 |
| **Histone DNA** | HCvsLC | -0.024 (-0.078, 0.031) | 0.39 |
|  | BMI | -0.001 (-0.006, 0.004) | 0.674 |
|  | COVID-19 infections (n) | 0.008 (-0.018, 0.033) | 0.558 |
|  | Medication usage | 0.007 (-0.003, 0.018) | 0.156 |
|  | Function | -0.000 (-0.000, 0.000) | 0.722 |
|  | Mental health | -0.017 (-0.074, 0.040) | 0.555 |
| **MPO DNA** | HCvsLC | -0.017 (-0.070, 0.035) | 0.515 |
|  | BMI | -0.000 (-0.005, 0.005) | 0.975 |
|  | COVID-19 infections (n) | -0.009 (-0.034, 0.015) | 0.451 |
|  | Medication usage | -0.001 (-0.010, 0.009) | 0.916 |
|  | Function | -0.000 (-0.000, 0.000) | 0.255 |
|  | Mental health | -0.004 (-0.059, 0.051) | 0.877 |

Data are presented for 51 patients with LC and 52 healthy controls. LC, long COVID; HC, healthy controls.
